# Ethics review of multi-centre trials in India: a survey of researchers and ethics committee members on perspectives, challenges, and opportunities

**DOI:** 10.1186/s12910-026-01489-1

**Published:** 2026-06-23

**Authors:** Nikhil Benroy Noronha, Jerin Jose Cherian, Gunjan Kumar, Tanu Anand, Sudipto Roy, Alka Turuk, Mayank Gangwar, Elna Paul Chalisserry, Sujata Sinha, Aruvi Poomali, Roli Mathur, Taruna Madan Gupta, Aparna Mukherjee

**Affiliations:** 1 Division of Development Research, ICMR HQ, New Delhi, India; 2 Department of Global Public Health, Karolinska Institutet, Stockholm, Sweden; 3 ICMR Bioethics Unit, Bangalore, India; 4 Department of Health Research, ICMR HQ, New Delhi, India; 5 Clinical Studies and Trials Unit, Division of Development Research, ICMR HQ, New Delhi, India

**Keywords:** Ethics committees, multi-centre trials, clinical research, India, common, Joint, Central, Designated, National, Regional review

## Abstract

**Background:**

While large multicentre trials (MCTs) helps in timely recruitment of participants and gives generalizable results, fragmented ethics approval processes delay trial initiation. This study explored the perspectives of ethics committee (EC) members and researchers on ethics review processes in MCTs, and approaches to streamline ethics approval.

**Methods:**

A cross-sectional survey with semi-structured and open-ended questions gathered perspectives (August–October 2024) from researchers and EC members across India using a non-probability, network-based sampling strategy. Survey items were developed based on study objectives and relevant literature, and organised into key domains on EC functioning, operational challenges, EC communication and alternative ethics review processes: joint, designated, and national/ regional ECs. Participants shared their perspective regarding the current and three hypothetical ethics review processes. Quantitative data were analysed descriptively; and open-ended responses underwent qualitative content analysis.

**Results:**

Of the respondents with MCT experience (106 researchers and 136 EC members), 21% of researchers and 31% of EC members supported the current model of independent EC review at each site. Less than half of respondents viewed the current process as very effective, and only about a quarter considered them efficient in avoiding delays. While researchers perceived designated ECs (30%) to have better efficiency, many EC members (32%) perceived joint reviews would ensure collaborative decision-making. National/ regional models were perceived to have challenges (16% EC members and 22% researchers) due to accountability concerns. Key challenges identified include absence of guidelines for joint review, conflicting decisions, need for training, prolonged timelines, resource constraints, and poor inter-EC communication.

**Conclusion:**

While there was no clear consensus for any of the proposed ethics review processes, consensus emerged regarding the need to optimise and reform the current process. These perspectives regarding operations of ECs could provide exploratory insights, although gathered using a non-probabilistic sample of potentially highly engaged respondents. These insights may inform further empirical research and discourse on contextually appropriate models to improving ethics review efficiency while safeguarding participant welfare.

**Clinical trial number:**

Not applicable.

**Supplementary Information:**

The online version contains supplementary material available at 10.1186/s12910-026-01489-1.

## Introduction

All clinical research involving human participants mandates independent ethics review and oversight, aimed at protecting participants’ health, rights, and overall well-being [1]. Ethics committees (ECs) are entrusted with reviewing research proposals and addressing potential ethical dilemmas within relevant sociocultural contexts. Such an independent review of proposals help identify and mitigate methodological and ethical concerns, and enhance the scientific value of research while ensuring safety of participants [2, 3]. For single-institution trials, the Institutional Ethics Committees of the host institution usually approves and handles the ethical oversight. In recent decades, the conduct of clinical research has progressively shifted from single-institution trials to coordinated, multi-site collaborations to meet the scientific and operational demands of modern clinical research landscape. Multi-Centre Trials (MCTs) are an effective means to enrol a large number of diverse participants in a timely manner. They offer a robust way to validate results across a range of demographic and geographic contexts, and improve the generalisability of findings [4–7]. However, in MCTs ethics approvals are typically required from the EC of each participating site. Timely completion, the major advantage of MCTs, is often lost due to the delay in obtaining separate approvals from ECs of individual participating study sites [8, 9]. 

These challenges are further compounded by systemic constraints faced by ECs including, high volume of research proposals, time/ budget constraints and administrative delays which may render the process highly bureaucratic [10–13]. Duplication of effort across multiple sites add to these existing delays, affecting trial initiation timelines [9, 14, 15]. 

This has led to discussions around reforming the way MCTs are reviewed and monitored by ECs [16–18]. However, empirical evidence from India on the experiences and challenges faced by ECs and researchers in the ethics review of MCTs remains limited [19]. In this context, researchers from the Clinical Studies and Trials Unit, and the Bioethics Unit of Indian Council of Medical Research (ICMR), undertook this study to explore the perspectives of Indian researchers and EC members. The study aimed to understand perspectives on the current review process and identify potential opportunities to streamline ethics review and approval procedures for MCTs. The purpose of the study was to guide future process reforms that could improve the efficiency and effectiveness of ethics reviews of MCTs in India.

## Methods

### Study design

The study was designed as a cross-sectional survey with semi-structured and open-ended questions.

### Recruitment of respondents

Non-probability sampling was employed and the survey was circulated between August-October 2024 via email to clinical researchers and ethics committee members known to have experience in conducting or reviewing MCTs. The invitation to participate was sent to researchers who are part of the Indian Clinical Trial and Education Network (INTENT), and EC members who are registered with the Department of Health Research (DHR), Government of India, and further requested to be snowballed to their relevant contacts and professional networks. It is mandatory for all institutions conducting biomedical research to register their ECs with DHR. INTENT is a nation-wide network of 84 clinical trial sites, including public and private medical colleges, hospitals, and research institutes. In total, 627 invitation emails were sent to researchers and 2406 to EC members. A reminder email was circulated once during the survey period to encourage participation.

Eligible participants included researchers and registered EC members who have been involved in MCTs. As there is no publicly accessible comprehensive database of all eligible researchers and EC members in the country, and owing to the snowballing strategy, the exact number of individuals who received the survey invitation and precise response rate cannot be ascertained.

### Survey instrument – development

Two survey instruments were developed using an abridged process to support content validity. They were designed to capture perspectives of EC members and researchers on ethics review processes for MCTs. The absence of a framework to assess the effectiveness of ethics reviews have been described earlier [20]. Hence, the authors have used a combination of literature review [8, 9, 15–18, 21] their own experience coordinating and reviewing MCTs, and independent subject-expert consultation to identify and refine items for the instrument. The items in the draft instrument were organised into predefined domains: perceived effectiveness and efficiency of ethics review, operational challenges, communication between ECs, and perspectives on alternative review models. Items included a mix of semi-structured and open-ended questions allowing for free-text space to share respondents’ perspectives and make reflective comments.

An independent bioethics expert (AS) reviewed the instrument for relevance, clarity, comprehensiveness, and alignment with the study objectives. Suggested revisions were minor and limited to refinement of wording to improve clarity. No formal pilot testing was conducted prior to survey dissemination. The expert review and refinement of instrument was considered appropriate to ensure adequate content validity for this exploratory survey.

Anonymised respondent information collected included type (researcher or EC member), sector (public or private), and level of experience with MCTs. To maintain confidentiality during data collection, no respondent identifier was collected and each response was assigned a unique code. The final survey tools used are available in Supplementary Material 1 & 2.

### Survey instrument – key domains

**Fig. 1:**
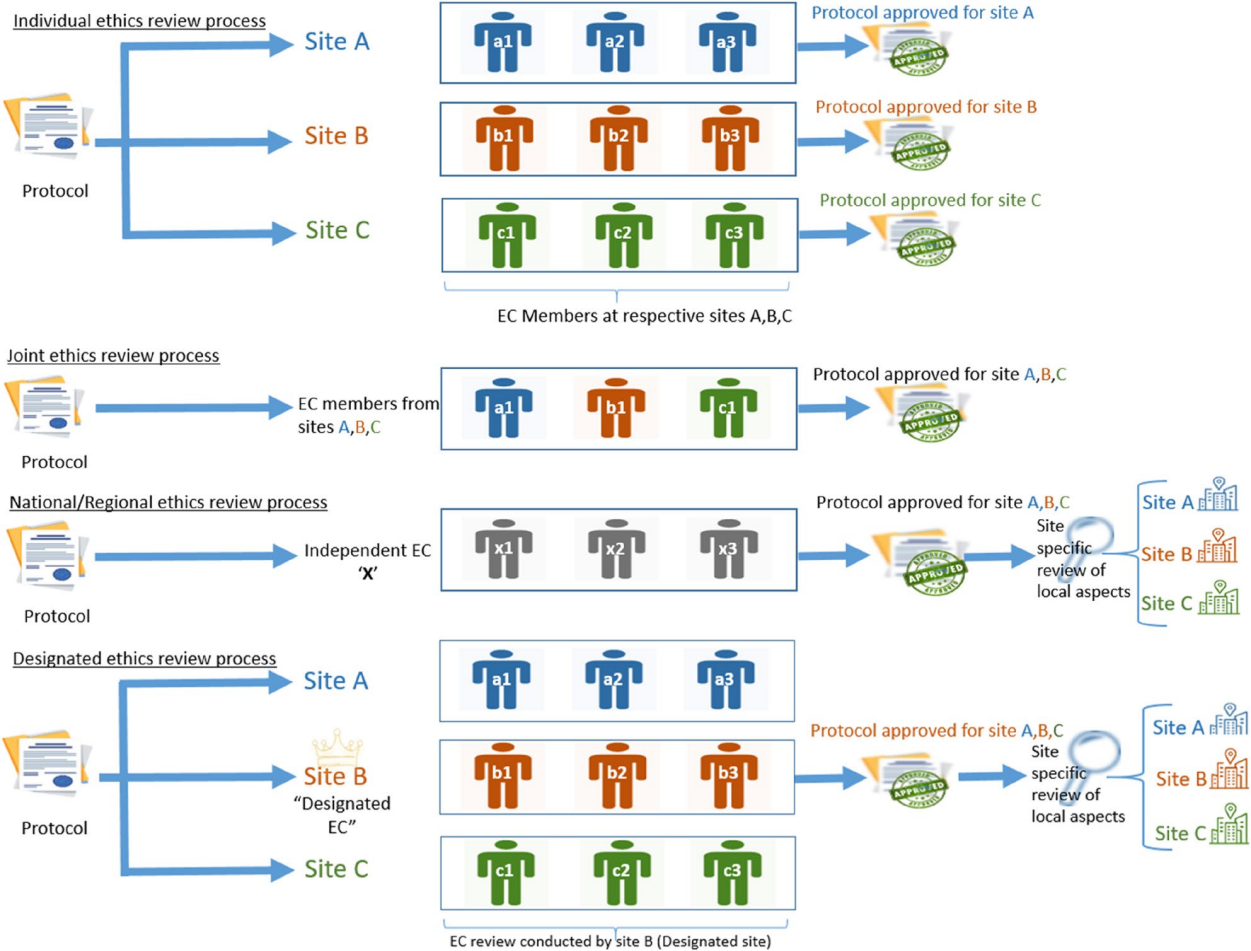
The current ethics review process, and three hypothetical processes – joint ethics review, national/ regional ethics review and designated ethics review. Source: Authors’ own illustration (created using Microsoft PowerPoint 2016)

The survey explored the following domains:

- Common perception regarding the functioning of ECs – For the purpose of this survey ‘effectiveness’ of an EC was defined as its ability in fulfilling the role of reviewing and monitoring an MCT, and ‘efficiency’ was defined as the time taken to review the proposals.
-  Operational challenges faced during review of MCTs.
- Communication between ECs.
- Process improvement – The perspective of respondents was also obtained regarding three hypothetical processes (Fig. 1 and Table 1) of ethics review of MCTs which were identified from suggestions in published literature [8, 9, 16, 17, 21], and internal discussion. The three processes identified were (a) Joint ethics review - Representative EC members from all participating centres of a MCT convene to collectively review the study proposal and arrive at a common decision. This process promotes harmonised EC decision-making across sites; (b) Designated ethics review - The EC of one participating site assumes the lead role in conducting the primary review of the MCT proposal, while ECs at other trial sites review the study with respect to locally relevant ethical and sociocultural considerations. This approach seeks to reduce duplication while preserving local contextual oversight; (c) National or Regional ethics review – An independent and dedicated EC constituted at national or regional level conducts the primary review of MCT proposal, and ECs at participating trial sites review only ethics aspects with local socio-cultural relevance This approach centralises review responsibility to enhance consistency and efficiency across sites.

**Table 1:** Critical activities of current and proposed ethics review processes

Various ethics review processes	Critical activities of ethics committees			
	Submission	Constitution of ethics committee	Review process	Approval process
Individual ethics review (current practice)	Researcher submits multi-centre research protocol separately to EC of each site	EC of each site is independently constituted as per prevailing institutional policy	The EC of each site reviews the research protocol asynchronously	The EC of each site provides an approval for study conduct only at their respective site
Joint ethics review	Researcher submits multi-centre research protocol to a common EC having representatives from ECs of all the participating sites	Representative EC members of all the participating sites in a multi-centre clinical trial convene together to review the protocol	The common EC reviews the research protocol together, considering both general ethics and site-specific aspects	The approval from a common EC should be sufficient for ECs of all the sites
Designated ethics review	Researcher submits multi-centre research protocol to one EC designated to review on behalf of all sites	One of the site ECs will be designated to review the general aspects of the protocol. The designated EC could be the PI’s EC or the EC with most experience etc.	The designated EC reviews the research protocol considering general ethics aspects only	The approval from a designated EC could facilitate the review by ECs of all the sites, as they now only need to review site-specific aspects
National / regional ethics review	Researcher submits multi-centre research protocol to national/ regional EC	The national EC will be constituted by reputed experts who will not be part of any of the site ECs where the study will be conducted	The national EC reviews the research protocol considering general ethics aspects only	The approval from a national EC could facilitate the review by ECs of all the sites, as they now only need to review site-specific aspects

### Ethical considerations

The study protocol was reviewed and approved by the ICMR-Central Ethics Committee on Human Research (ICMR-CECHR 003/2024, dated 08/06/24). The STROBE reporting guideline [22] informed the reporting, and the STROBE checklist [23] is provided as Supplementary Material 3.

### Data analysis

Data sets were analysed using Microsoft Excel (2016) for Windows. Quantitative data were analysed using descriptive statistics (proportions or means), and no adjustments for non-probability sampling were applied. Responses from open-ended questions were analysed using qualitative content analysis. Following familiarisation with the responses, open coding lead to preliminary codes being assigned to perspectives of respondents. Similar codes were grouped into broader categories through iterative discussion among three authors (NBN, JJC, and GK). Categories were refined to improve conceptual clarity and alignment with study objectives. Differences in interpretation were resolved through consensus. Final categories were used to summarise recurring perspectives within the data.

Responses to closed questions were mandatory in the survey instrument; therefore, there were no missing data for these variables. We additionally performed subgroup analysis based on affiliation and experience of the responders to understand differences in perspective.

## Results

### Respondent characteristics

Among the 3,033 directly invited participants, responses were received from 149 researchers and 207 EC members, yielding a primary response rate of 11.7% based on the directly reachable denominator. 71% researchers (106 respondents) and 66% of the EC members (136 respondents) reported prior experience with MCT. Only these responses were considered for further analysis. Among the 136 EC members, 70% (*n* = 95) were affiliated with private sector institutions, while among the 106 researchers, 56% (*n* = 59) were affiliated with public sector.

Nearly half of the respondents had more than five years of experience in MCTs, and about 58% of the EC members and 28% researchers were involved in more than five MCTs in the past five years. The respondents’ prior involvement in coordinating, conducting, or reviewing MCTs is summarised in Table 2. No missing data were observed for closed-ended variables, as responses were mandatory.

**Table 2:** Experience of the respondents in handling MCTs

		Number of responses; n(%)	
		Researchers Total (*N*) = 106	EC members Total (*N*) = 136
Years of experience in coordinating^†^/ conducting^†^/ reviewing^¥^ MCTs	1–5 years	52(49%)	72(53%)
	5–10 years	22(21%)	33(24%)
	10–15 years	13(12%)	18(13%)
	> 15 years	19(18%)	13(10%)
Number of MCTs coordinated^†^/ conducted^†^/ reviewed^¥^ (Last 5 years)	1–5 trials	78(74%)	78(57%)
	5–10 trials	20(19%)	26(19%)
	10–20 trials	5(5%)	18(13%)
	20 + trials	3(2%)	14(10%)

^†^ Experience of researchers in coordinating/ conducting MCTs were collected

^¥^ Experience of EC members in reviewing MCTs were collected

### Perceived functioning

Only 45% researchers and 51% EC members considered ECs to be very effective in fulfilling its role, and 22% researchers and 27% EC members considered their ECs to be efficient in avoiding delays. Both groups agreed that the common topics discussed during the ethics review of a MCT included reviewing clinical trial designs, protocols, and ancillary documents, informed consent procedures, adverse events, and compliance with ethics guidelines and regulations. Perspectives of respondents on the effectiveness, efficiency and common issues discussed by ECs in MCTs are outlined in Table 3.

**Table 3:** Perceptions of researchers and EC members regarding effectiveness, efficiency and common issues discussed by ECs pertaining to MCTs

		Number of responses; n(%)	
		Researchers Total (*N*) = 106	EC Members Total (*N*) = 136
EC Effectiveness in MCT Review and Monitoring	Very effective	48(45%)	70(51%)
	Somewhat effective	42(40%)	46(34%)
	Neutral	7(7%)	15(11%)
	Not very effective	7(7%)	5(4%)
	Unsure/Unable to comment	2(1%)	0(0%)
EC Protocol Review Time for MCT	Consistently meets timelines without significant delays	23(22%)	37(27%)
	Usually meets timelines, occasional minor delays that do no impact the trial	39(37%)	64(47%)
	Meets timelines, but sometimes delays occur which have minor impact on the trial	31(29%)	28(21%)
	Frequent delays impacting timelines of the trial significantly	12(11%)	6(4%)
	Not aware	1(1%)	1(1%)
Common issues discussed during the ethics review of a MCT^†^	Reviewing the clinical trial design and methods of a MCT study protocols and ancillary documents	83(78%)	114(84%)
	Overseeing informed consent procedures	71(67%)	93(69%)
	Review of adverse events	68(64%)	88(65%)
	Ensuring compliance with ethics guidelines and regulations	63(58%)	87(64%)
	Addressing data sharing, ownership of materials and data, intellectual property rights (IPRs), and joint publications.	60(57%)	70(51%)
	Recommendation of compensation for trial related injury/ death	55(52%)	68(50%)
	Managing conflicts of interest – including MOUs coverage standardization	55(52%)	68(50%)
	Review of local socio-cultural aspects of a clinical trial	42(40%)	63(46%)
	Monitoring of the respective EC’s participating site of MCT	46(43%)	59(43%)
	Causality assessment of SAE	46(43%)	60(44%)
	Engaging with the regulator for submission of SAE related information	35(34%)	54(40%)
	Others (inter-institutional considerations, sponsor credibility, non-allopathic trials)	8(8%)	3(2%)

^†^ Multiple responses allowed from respondents

### Operational challenges

The challenges faced by ECs and researchers while reviewing or monitoring MCTs included limited resources, high workloads and insufficient training or expertise (Table 4). Another key challenge identified was the lack of a formal mechanism to harmonize decisions and share critical information (SAEs, monitoring updates, protocol deviations) with other sites’ ECs, making inter-site coordination and monitoring difficult. Conflicting EC recommendations, varying timelines across different ECs, inconsistent capacity among ECs, and documentation burden, were the challenges faced by researchers while submitting a MCT protocol for IEC approval. Details are provided in Supplementary Material 4.

**Table 4:** Common practices and key challenges during ethics review of MCT

		Number of responses; n(%)	
		Researchers Total (*N*) = 106	EC Members Total (*N*) = 136
Communication with ECs of participating sites	Yes	30(28%)	22(16%)
	No	76(72%)	95(70%)
	I don’t know	0(0%)	19(14%)
Common topics for communication with other ECs^†‡^	Protocol deviation	20(67%)	16(73%)
	SAE	22(73%)	14(64%)
	Adverse events	15(50%)	13(59%)
	Progress of enrolment	14(47%)	13(59%)
	Compensation for trial related injury	14(47%)	10(45%)
	Others (Data Safety)	0(0%)	1(5%)
Challenges observed by EC for effective review and monitoring of MCTs^†^	Limited Resources	55(52%)	68(50%)
	Difficulty in harmonizing recommendations with other ECs	45(42%)	44(32%)
	High workload leading to untoward delay	46(43%)	34(25%)
	Insufficient training or expertise	35(33%)	47(35%)
	Lack of standardized procedures	35(33%)	32(24%)
	No challenges	13(12%)	24(18%)

^†^ Multiple responses allowed from respondents

^‡^ Of the 30 researchers and 22 EC members who believe that EC of the main site should communicate with the ECs of other participating sites

### Communication between ECs

76% researchers and 82% EC members believed that an approving EC of one site should communicate with ECs of other participating sites, and provide extracts of EC decisions.

### Process improvement

86% researchers and 89% EC members opined that the decision-making of ECs could be improved through the introduction of new guidelines, while 94% EC members expected special training to improve the situation.

**Fig. 2:**
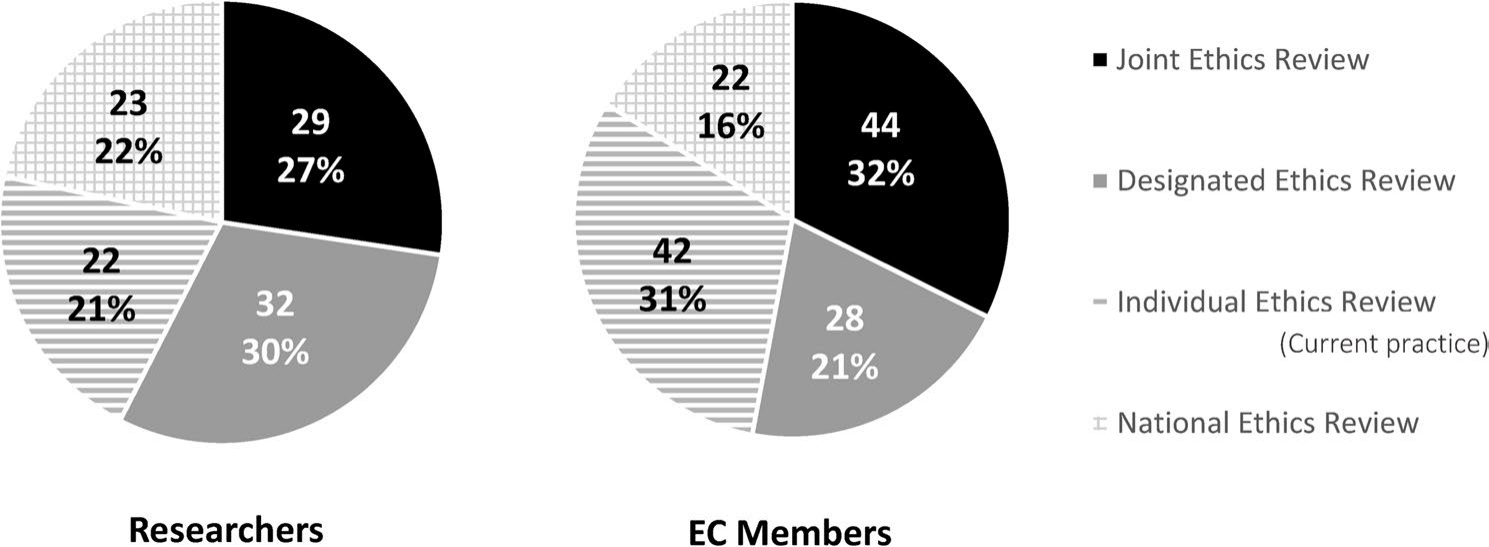
Most effective common ethics review process in the perspectives of researchers and EC members

Only 21% of researchers and 31% of EC members wished to continue with the current practice of separate ethics review of all participating sites of an MCT (status quo). While choosing an alternate process, 30% of the researchers favoured a designated ethics committee and 32% of the EC members preferred a joint ethics review. Only 22% researchers and 16% EC members supported regional or national ethics review committees (Fig. 2). When analysed on the basis of the respondents’ affiliation and years of experience, the choices did not differ significantly between private and public researcher’s/ EC members or between researcher’s /EC members with more than 10 years of experience vs. the less experienced ones. Subgroup analysis of perspective based on affiliation and experience of the responders are provided in Supplementary Material 5–7.

Outcome of EC reviews of MCTs when they have been reviewed earlier by a common/ designated/ national or regional EC were analysed to understand the subsequent steps. Only 25% researchers and 18% EC members had faced such situations. Of them, 56% researchers and 14% EC members faced subsequent review. The lack of a clear process to identify the designated EC was also recognised. The suggested criteria based on which a designated EC may be selected is given in Supplementary Material 8. Perspectives concerning the responsibilities of EC reviews of proposals that have already been reviewed by joint/designated/national EC are described in Supplementary Material 9 and the foreseeable challenges for each of the ethics review processes are given in Table 5.

**Table 5:** Foreseeable challenges for various ethics review processes

Foreseeable challenges for various ethics review processes								
Joint Ethics Review			Designated Ethics Review			National/Regional Ethics Review		
Challenges	Researchers	EC members	Challenges	Researchers	EC members	Challenges	Researchers	EC members
Logistic issues to plan/ conduct the meeting	4(4%)	93(68%)	There is no clear process/ criteria to identify the designated ethics committee	56(53%)	62(46%)	There is no clear process to constitute the national ethics committee	60(57%)	72(53%)
Monitoring of the trial will continue to be the responsibility of the site EC, which was not fully involved in the decision making process	70(66%)	91(67%)	Decision making can seem unilateral and might not be agreeable to the local ECs	76 (72%)	92(68%)	Decision making can seem unilateral and might not be agreeable to the local ECs	65(61%)	77(57%)
Disagreements between members are difficult to resolve	67(63%)	76(56%)	Monitoring of the trial will continue to be the responsibility of the site EC, which was not fully involved in the decision making process	59(56%)	74(54%)	Disproportionate amount of workload and untoward delays if a limited number of national/ regional ethics committees will have to review all MCTs	59(56%)	67(49%)
Difficulty to identify the chairperson and member secretary	42(40%)	44(32%)	Inability of designated EC to monitor all sites physically	60(57%)	80(59%)	Monitoring of the trial will continue to be the responsibility of the site EC, which was not fully involved in the decision making process	59(56%)	81(60%)
Every trial will end up having a new EC	47(44%)	43(32%)	No challenges	1(1%)	3(2%)	Inability of national/ regional EC to monitor all sites physically	56(53%)	78(57%)
Accountability and Responsibility	3(3%)	1(1%)	Others	5(5%)	1(1%)	No challenges	1(1%)	4(3%)
Others	3(3%)	2(1%)	-	-	-	Others	3(3%)	1(1%)

### Categories from qualitative content analysis

Open-ended responses were grouped into categories related to perceived advantages of different ethics review models. The findings are given below as descriptive categories, with abridged verbatims to support the authors’ interpretative claims.

Category 1 - Efficiency: Researchers and EC members considered efficiency and process streamlining as key advantages for joint and designated ethics review processes.

“*Saves a lot of time and prevents delays*” (EC Member 2).

“*Designated Ethics Committee review followed by an expedited review at participating EC will save a lot of time*,* effort and paperwork”* (Researcher 13)..

“*It will save time and resource utilization”* (Researcher 18).

Interpretive claim: Efficiency was a common reason for preference of alternative ethics review models, particularly those reducing sequential review processes.

Category 2 - Consistency: Respondents considered joint and national/ regional ethics review committees to promote uniformity in practices and reduction in conflicting recommendations.

“*This can help to harmonize the ethics review process. Regional ethics committees can review documents for independent researchers and guide institutional ethics committees*” (EC Member 7).

“*Common ethics review will help in making shared decision”* (EC Member 17).

Interpretive claim: Respondents opined that improved collaboration across ECs can improve shared decision-making.

Category 3 - Expertise: National/ regional ethics review committees were perceived to enable access to experts with better experience and specialised expertise.

“*A sensible option is to have centralised ethics review*,* as IECs across institutions may vary in capacity and potential biases*” (Researcher 13).

Interpretive claim: Some respondents perceived that centralised ethics review processes can improve the quality of review by pooling expertise.

Category 4 – Local context: Some respondents supported the current system of ethics review at each of the individual sites since it best addresses local socio-cultural factors and is most capable of effective oversight.

“*Different sites may have different patient populations. Some issues may be specific to certain sites and are better reviewed by the IEC of that site*” (Researcher 9).

“*I uphold the current practice of full review of multicentre trial protocols by each participating site’s ethics committee*,* so that local social or cultural issues related to study participants can be addressed” (*EC Member 18).

Interpretive claim: Some respondents valued decentralised ethics reviews despite of its inefficiencies, due to its perceived ability to uphold contextual sensitivity.

A tabular summary of the advantages of various ethics review processes based on the responses from EC members and researchers are provided in Supplementary Material 10.

## Discussion

Evidence Gap: While literature regarding the roadblocks to conducting efficient MCTs abound [9, 24–26], the nuances of ethics review process of MCTs, in the Indian context, is sparse. Most of the literature from India describe the challenges in ethics review process from a single EC or focus on the perspectives of either researchers or EC members, and don’t specify if trials were single or multi-centre [27–32]. Our study included perspectives from both researchers and EC members involved in MCTs across all states in India. Although the study includes respondents from multiple institutions and regions, the use of non-probabilistic sample of possibly highly engaged respondents limits its generalizability. The perspectives captured are therefore likely to reflect those of researchers and ethics committee members with a keen interest in ethics governance, rather than a representative cross-Sect [33]. 

Perceived functioning: Our survey found that 85% of both EC members and researchers considered ECs to be generally effective. This is consistent with the findings of Dakhale et al. that ECs play a crucial role in adhering to ethical standards, improving procedural rigor and safeguarding research participants [29]. About three-fourths of our respondents reported delays that impacted their timelines. In an MCT, delay in receiving approval from even one institutional EC can adversely affect timelines [9]. There can be multiple reasons for this, including variability in the review process [25, 34–36], inherent structure of the review process [9, 32], inefficiencies on the part of researchers or EC members [37], and inadequate resources, which have been widely reported as a challenge in clinical trials and are often amplified in MCTs [11, 13, 38]. These responses provide exploratory insights regarding effectiveness or efficiency within the study sample, but may not be generalisable to other settings.

While the National Ethical Guidelines for Biomedical and Health Research Involving Human Participants by ICMR, 2017 and the New Drugs and Clinical Trial Rules (NDCT) by Central Drugs Standard Control Organisation (CDSCO), 2019 [16, 39] have strengthened the framework for EC reviews, ECs continue to face numerous challenges in protocol review of MCTs. A previous study by ICMR reported that ECs reviewed more than sixty projects per meeting, indicating a high workload [40]. Additional challenges include lack of trained manpower, infrequent meetings, administrative challenges, inadequate infrastructure, and budget constraints [24, 41–43]. In line with previous literature, our study also emphasised the need for capacity building and process optimisation [9, 24, 30, 44]. Interestingly, 12% of EC members and 18% of researchers reported facing no challenges, implying diversity in processes across various institutions.

In line with previous work, 72% of researchers and 70% of EC members in our study reported poor communication between the various ECs in an MCT as a challenge, highlighting systemic inefficiencies in MCT coordination [24, 30]. Research conducted globally highlights significant variations among ECs and even among individual members of the same committee when evaluating the same protocol [35, 45, 46]. Our study identified that 44% of researchers and 32% of EC members faced conflicting EC recommendations while being part of an MCT.

IEC review preferences: The respondents’ perceptions suggest that the current system of individual site EC review of MCTs offers an advantage in addressing the local socio-cultural context, site-specific requirements, and inclusivity at the local level. This benefit was strongly emphasized by respondents who favoured the decentralized ethics review system. Other notable advantages in the opinion of the respondents include the accountability and ownership of decisions, which rest with the local EC. A proportion of the EC members believed that multiple separate review would improve the rigour of ethical oversight. However, as reported previously, our respondents also reported that separate reviews were associated with extended timelines and duplication of effort [47]. Our findings highlight that the current practise of individual site EC review was preferred by less than 1/3rd of researchers and EC members, suggesting the possibility of considering alternate review processes. This result is supported by work of previous authors [9, 32, 43] Our respondents also believed that post-approval responsibilities such as safety reporting, SAE assessment, compensation determination and monitoring would continue to remain the responsibility of the site investigator and the local EC.

Recognising the shortcomings of the current ethics review process, an important subsequent question is which alternative approach would researchers and EC members consider useful. Across the three hypothetical ethics review processes suggested to the respondents, the preferences of respondents were distributed with no clear consensus. The nature of affiliation (public vs. private institute) or years of experience with MCTs (> 10 years vs. 1–10 years) did not significantly affect the response.

Firstly, the joint ethics review was commonly perceived by both researchers and EC members, to reduce delays and shorten the timelines. Respondents perceived that a joint EC could enhance collaboration and consistency in ethical standards. It allows for diverse perspectives from representatives of multiple ECs, ensuring that the unique cultural and ethical considerations of each centre are addressed. It also solves the problem of conflicting opinions across different participating committees.

Secondly, for designated ethics review, a lead institute was perceived to improve timelines, and help standardise processes, although it may deter collaborative decision-making.

Finally, national/ regional ethics review was perceived by some respondents to improve standardisation, streamlining, and robust evaluation, though many protocols still required local EC review, underscoring the need for local oversight. Studies report similar observations about centralised ethics review processes including loss of local context or site-specific relevance, loss of revenue to the local ECs, uncertainty about the quality and expertise of the reviews, and its unsure impact on the local community [13, 32, 48]. 

No single review process had a clear consensus among the studied sample. However, perspectives of the respondents provide useful exploratory insight into perceived advantages of various ethics review processes. These findings should be interpreted as exploratory and hypothesis-generating, that could trigger further research.

Ethics review processes in other countries: Various countries have identified this challenge and tried to address it differently. Some countries have made a shift from decentralised ethics review for MCTs to alternate ethics review processes [18, 26]. Australia (via the National Mutual Acceptance [NMA] Scheme), the UK (via the Health Research Authority [HRA] and Research Ethics Committees [REC]), the US (under the Revised Common Rule [2018]), and EU member states (via the EU Clinical Trials Regulation [CTR] 536/2014) have adopted alternative models meant to streamline MCT ethics approvals through single-site or centralised ethical review systems followed by local governance approvals at the site level [49–52]. The two step process, with the second site-specific assessment assures the site-specific feasibility, resource availability and compliance with relevant laws at the sites, overall enhancing the participant safety, improving timelines while maintaining ethical standards [53]. 

### Strengths

Our study presented perspectives from both EC members and researchers from various geographies and institutional settings across India, while maintaining respondent anonymity. Majority of the respondents were experienced in reviewing or conducting MCTs. The perspective on the current ethics review process for MCTs was captured, while reflecting on potential hypothetical ethics review processes for a harmonised national framework, offering exploratory insights to inform future discourse.

### Limitation.

A limitation of this study is the use of snowball sampling, which prevents accurate sample estimation since secondary redistribution is untraceable. Hence, the denominator used for estimating response rate was limited to directly invited participants. This strategy may have introduced self-selection and network biases, leading to an overrepresentation of individuals highly engaged in research governance, and limiting divergent perspectives. This can affect the generalizability of results since respondents are likely to have a keen interest in ethics governance, and may not be representative of the broader population of researchers or EC members in India. However, the study remains useful in providing exploratory insights from informed stakeholders on perceived challenges and potential opportunities to improve ethics review processes for MCTs.

We acknowledge that qualitative insights derived from brief open-ended responses in an online survey inherently lacks depth, as compared to data generated through interviews or focus groups. However, these items were included a priori as an adjunct to the primary survey to capture perspectives that may not be adequately reflected in fixed-response options. As described by LaDonna et al., [54] free-text responses can provide valuable inputs when they address specific exploratory research questions and are analysed proportionate to the nature of the data. These findings should be interpreted as descriptive supplementary insights that can feed future research, rather than in-depth qualitative accounts.

As the survey was anonymous, respondents with dual roles could not be identified, and some overlap between researcher and EC responses are expected. The focus was on MCTs, hence no distinction was made between industry-sponsored and investigator-initiated trials. Additionally, the survey instrument did not undergo formal pilot testing before circulation due, which may have affected its validity.

## Conclusions

In conclusion, this study offers a perspective of both researchers and EC members on the ethics review landscape for MCTs in India. These perspectives regarding operations of ECs from a non-probabilistic sample, could provide exploratory insights, even though they may not be generalizable as the perspective of Indian researchers or EC members. While majority of respondents acknowledged the general effectiveness of ECs, significant concerns regarding timelines were identified. Our data highlights challenges of MCTs ranging from variability in review processes and resource constraints, to communication gaps between ECs at different sites. Our study emphasized the need for process optimization to improve ethics review of MCTs.

Although a clear consensus on an alternative review process did not emerge, the need for an alternate process was highlighted since less than 1/3rd of responders favoured the current process. The data reveals exploratory insights into the perceived advantages and disadvantages of individual, joint, designated, and national ethics review processes. Our findings underscore the complexity of optimizing MCT ethics review in India and could provide insights to help develop contextually appropriate and efficient process that balances local needs with the overarching goal of protecting participant interests. These insights may contribute to ongoing discussions and future research meant to improve EC efficiency while maintaining robust ethical oversight.

## Supplementary Information

Supplementary Material 1.

## Data Availability

The dataset generated and analysed during the current study is anonymous. The data are available from the corresponding author upon reasonable request.

## Abbreviations

DHR: Department of Health Research
EC: Ethics Committee
ICMR: Indian Council of Medical Research
INTENT: Indian Clinical Trial and Education Network
MCT: Multi-centre trial
