# Supplementary material for: Ethics review of multi-centre trials in India: a survey of researchers and ethics committee members on perspectives, challenges, and opportunities
Source: BMC Med Ethics. 2026 Jun 23;27:119. doi: 10.1186/s12910-026-01489-1 (PMC13308206; doi:10.1186/s12910-026-01489-1)
Supplement: Supplementary file 1 — Supplementary Material 1. [file 12910_2026_1489_MOESM1_ESM.docx]

Supplementary Material

## Supplementary Material 1

Google form questionnaire to researchers

**Ethics Review of Multi-Centre** **Clinical Trials: Researcher Perspective**

**Trial Methodology Research by Indian Clinical Trial And Education Network**

**(INTENT)**

Investigators and Contact Information

Dr Aparna Mukherjee (*Principal investigator*), Clinical Studies and Trials Unit, Division of Development Research, Indian Council of Medical Research, New Delhi. (aparna.sinha.deb@icmr.gov.in)

Dr Jerin Jose Cherian (*Co-Investigators*), Clinical Studies and Trials Unit, Division of Development Research, Indian Council of Medical Research, New Delhi

(cherian.jj@icmr.gov.in)

Dr Gunjan Kumar (*Co-Investigators*), Clinical Studies and Trials Unit, Division of Development Research, Indian Council of Medical Research, New Delhi

(gunjank.hq@icmr.gov.in)

**Problem Statement:** Multicentre clinical research are most useful in situations where we need to recruit patients in large numbers, for rare diseases or want to have better generalisable results. However they have various challenges in its planning and conduct. Ethics review of multicentre studies are known to be cumbersome, since the current accepted method is to obtain ethics clearance separately from individual study site. In the interest of improving the Indian clinical research ecosystem, there are various other methods to streamline ethics clearance which are being explored in this survey.

**Invitation to participate in the survey:** You are being invited to participate in this survey to invite your perspectives on ethics review processes. Your expertise in the conduct of multi-centre clinical trials will prove extremely useful to understand the current challenges and solutions. This is part of a trial methodology research conducted by ICMR's INTENT team in the study titled “Ethics Review of Multi-centre Clinical Trials: An Indian Perspective". The INTENT program of ICMR is positioned to conduct large multi-centre clinical trials to explore solutions for diseases of national health priority. Streamlining the ethics review process will help INTENT by reducing redundant processes and avoiding delays.

Please note that this form is meant to be filled in if your primary role is that of an Indian researcher/ investigator in a multi-centre clinical trial. However, if your primary role for the purpose of this survey is that of an ethics committee member, then please use https://forms.gle/y4Kwqo7VzaGDScyk8

The survey will take less than fifteen minutes.

**Goal of the survey:** This study aims to understand the perspectives of researchers and ethics committee members regarding ethics review processes in multi centre clinical trials. Your participation in this survey to clearly understand the Indian system for ethics evaluation of multi-centre clinical trials will be invaluable. Before agreeing to participate in this study, it is important that you read and understand the following information.

**Objectives:**

This survey has three objectives:

1) To understand the challenges faced by researchers in obtaining approval from ethics committees for multi-centre trial

2) To understand the perspectives of ethics committee members regarding the review of multi-centre trials **(**[**different survey**](https://forms.gle/y4Kwqo7VzaGDScyk8)**)**

3) To find out the possible solutions to streamline the ethics review of multi-centre clinical trials

**Study design:** This is a brief, anonymous, national-level, cross-sectional survey administered using Google Forms. We are inviting researchers, affiliated with diverse types of institutions and organizations from India. We will also reach out to chairpersons/ members of ethics committees

to understand their perspectives on these matters using a separate survey.

**Consent:**

We thank you for agreeing to participate in this survey. Your feedback will help us understand the current practices and challenges faced by ethics committee members and researchers in evaluating multi-centre clinical trial protocols and monitoring such clinical trials. Please answer the following questions to the best of your knowledge. The survey will take less than ten minutes. Filling up this survey form will be considered as your consent to use the information provided. There are no identifiers **(no details of participants are collected - including email address)** that are being collected in the survey, and responses will be analysed in a pooled manner.

**Acknowledgement of contribution:** The results of this survey will be used to improve clinical trial ecosystems by streamlining the ethics review process. We will acknowledge the participants of the survey in the manuscript if allowed and the data gathered from this survey will be anonymised before it is published. Please be aware that you will not be receiving any other compensation for participating in this survey.

**Conflict of Interest:** The members of our research team declare that they have no conflict of interest and will receive no financial benefits from any source based on the findings of this survey. Our study does not receive any sponsorship or funding from any source.

**Questions About the Study:** If you have questions about this research study, please contact any of the investigators mentioned earlier.

* Indicates required question

# Participant details

1. (1) Are you a researcher/ investigator with experience in the planning orconduct of multi-centre clinical trials? *

*Mark only one oval.*

Yes

No *Skip to section 5 (****Thank you****)*

# Participant details

This section will collect general information about participant and their institution. No identifiers will be collected.

1. (2) Nature of your affiliated institution * *Mark only one oval.*

Public sector teaching hospital

Private sector teaching hospital

Public sector non-teaching hospital

Private sector non-teaching hospital

Industry/ CRO

Independent (standalone) research institute

Independent (standalone) ethics committee

Other:

1. (3) Which Indian state is your affiliated institution located in * Dropdown

*Mark only one oval*

1. (4) Which of the following is the registration status of your *

institutional ethics committee (IEC) *Mark only one oval.*

Dropdown

Both DHR and CDSCO registered

CDSCO registered but not DHR registered

DHR registered but not CDSCO registered

Neither DHR nor CDSCO registered

I don't know

1. (5) How many years of experience do you have in coordinating/leading multi-centre clinical trials as an investigator or researcher? *

Dropdown

*Mark only one oval.*

1-5 years

5-10 years

10-15 years

>15 years

| 6. | (6) Since 2019, how many multi-centre clinical trials have you coordinated/ led as an investigator or researcher*dropdown  *Mark only one oval.* |
| --- | --- |

1-5

5-10

10-20

>20

1. (7) In your opinion, how effective is your IEC in fulfilling its role in review and monitoring of a multi centre clinical trial? *

*Mark only one oval.*

- - 1. Very effective
    2. Somewhat effective
    3. Neutral
    4. Not very effective
    5. Not at all effective
    6. I don't know

1. (8) In your opinion, what could be the challenges faced by your IEC for effective review and monitoring of multi centre clinical trials? * (multiple responses allowed)

*Tick all that apply.*

- 1. Limited resources (e.g., funding, personnel)
  2. Insufficient training or expertise
  3. High workload leading to untoward delay
  4. Lack of standardized procedures
  5. Difficulty in harmonizing decisions with other committees
  6. No challenges

Other:

1. (9) What are the common issues discussed during the ethics review of a multicentre clinical trial. (Multiple responses allowed) *Tick all that apply*Reviewing the clinical trial design and methods of a multi centre clinical trial study protocols and ancillary documents

Overseeing informed consent procedures

Review of adverse events

Causality assessment of serious adverse events

Recommendation of compensation for trial related injury/ death

Ensuring compliance with ethics guidelines and regulations

Monitoring of the respective IEC's participating site of multi centre clinical trials

Engaging with the regulator for submission of SAE related information

Review of local socio-cultural aspects of a clinical trial

Managing conflicts of interest – including MOUs coverage standardization

Addressing Data sharing, ownership of materials and data, intellectual property rights (IPRs), and joint publications.
Other:

10. (10) How would you rank the average time taken by your Ethics committee to review multi-centre clinical trial protocols? *

*Mark only one oval.*

a)Very efficient (Consistently meets timelines without significant delays)

b)Generally efficient (Usually meets timelines, occasional minor delays that do

no impact the trial)

c)Adequate (Meets timelines, but sometimes delays occur which have minor

impact on the trial)

d)Inadequate ( Frequent delays impacting timelines of the trial significantly) E)Not aware

# For Researchers/ Investigators of multi-centre clinical trials

This section of the survey tries to understand the perspectives of researchers/ investigators regarding the ethics review of multi-centre trials. We also explore potential solutions to streamline the ethics review of multi-centre clinical trials.

1. (11) Are you aware of the draft guidelines for Common Ethics Review of Multicentre Research by ICMR that is currently published for public consultation inviting comments from relevant stakeholders? *

*Mark only one oval.*

Yes

No

1. (11) a. If ‘YES’ to the previous question, Are there any specific areas or issues unaddressed in the ICMR guidelines? (If ‘NO’ to previous question, kindly skip)
2. (12) Do you think that a guideline for ethics review of multicentre trials would aid ethics committees in their decision making? *

*Mark only one oval.*

Yes

No

Not Sure

1. (13) Although it is not mandatory for investigators to provide extracts of Ethics Committee (EC) decisions from other participating centres for multi-centre trials, do you believe it should become a regular practice and part of the guideline? *

*Mark only one oval.*

Yes

No

1. (14) What are the challenges that you have faced as a researcher/ investigator in obtaining ethics clearance for protocols of multi-centre clinical trials (multiple responses allowed)

*Tick all that apply.*

Varying timelines across different IECs

Conflicting recommendations/decisions across different IECs

Inconsistent capacity across different IECs

Documentation burden Other:

1. (14) (a) Please justify the response chosen in question 10 (In up to 200 words) *
2. (15) As a researcher /Investigator of a multi-centre clinical trial, besides communicating with your local IEC, do you communicate with IECs of other participating sites*

*Mark only one oval.*

Yes

No

1. (15) (a) If 'Yes' in previous question, what topics do you find necessary to communicate (multiple responses allowed) (If 'NO' Kindly skip this question)

*Tick all that apply.*

Adverse events

Serious adverse events

Progress of enrolment

Protocol deviation

Compensation for trial related injury
Other:

1. (16) As an Investigator, how often have you come across conflicting ethics committee recommendations/decisions for a multicentre clinical trial? *

*Mark only one oval.*

Always

Often

Rarely

Never

1. (17) In your opinion, which of the following processes would be most effective in improving the ethics review of a multi-centre clinical trial protocol (choose only one most appropriate response) *Mark only one oval.* *

Common ethics review (when representative IEC members of all the participating centres in a multi-centre clinical trial convene together to review the protocol)

Designated ethics committee (when one of the participating centres takes the lead role to review the protocol of a multi-centre clinical trial and their recommendations are sufficient for the IECs of remaining trial sites to allow the conduct of the trial after review of ethics aspects with local socio-cultural relevance)

National or regional ethics committee (when a dedicated ethics committee is constituted to review multi-centre clinical trial protocols and their recommendations are sufficient for the IECs of remaining trial sites to allow the conduct of the trial after review of ethics aspects with local socio-cultural relevance)

Full ethics review from each participating site's IEC, as is practiced currently

1. (17) (a) Please justify the response chosen in question 17 (mandatory, in up to

200 words) *

1. (17) (b) From the following challenges, which are the ones that will be faced in case of implementation of **common ethics review** (multiple responses allowed)

[Common ethics review = when representative IEC members of all the participating centres in a multi-centre clinical trial convene together to review the protocol]

*Tick all that apply.*

Disagreements between members are difficult to resolve

Every trial will end up having a new ethics committee

Difficulty to identify the chairperson and member secretary

Monitoring of the trial will continue to be the responsibility of the site IEC, which was not fully involved in the decision making process

Other:

1. (17) (c) From the following challenges, which are the ones that will be faced in case of implementation of **designated ethics committee*** (multiple responses allowed)

[Designated ethics committee = when one of the participating centres takes the lead role to review the protocol of a multi-centre clinical trial and their recommendations are sufficient for the IECs of remaining trial sites to allow the conduct of the trial after review of ethics aspects with local socio-cultural relevance]

*Tick all that apply.*

There is no clear process/ criteria to identify the designated ethics committee

Decision making can seem unilateral and might not be agreeable to the local IECs

Monitoring of the trial will continue to be the responsibility of the site IEC, which was not fully involved in the decision making process

Inability of designated ethics committee to monitor all sites physically

Other:

24. (17) (c1) In your opinion, if a **designated ethics committee** were to review the protocol of a multi-centre clinical trial, what would be the criteria based on which such a designation would be assigned (choose only one most appropriate response)

[Designated ethics committee = when one of the participating centres takes the lead role to review the protocol of a multi-centre clinical trial and their recommendations are sufficient for the IECs of remaining trial sites to allow the conduct of the trial after review of ethics aspects with local socio-cultural relevance]

IEC of the institute of lead PI can be designated

IEC with maximum experience in review of clinical trials, especially multi centre clinical trials can be designated

IEC with closest date available amongst the various sites can be designated

IEC scoring highest in an objective evaluation tool, such as the WHO Tool for

Benchmarking Ethics Oversight of Health-Related Research can be Designated

IEC with the least rejection rate can be designated

Other:

25. (17) (d) From the following challenges, which are the ones that will be faced in case of implementation of **national or regional ethics committee** (multiple responses allowed) *

National or regional ethics committee = when a dedicated ethics committee is constituted to review multi-centre clinical trial protocols

*Tick all that apply.*

There is no clear process to constitute the national or regional ethics committee

Decision making can seem unilateral and might not be agreeable to the local IECs

Monitoring of the trial will continue to be the responsibility of the site IEC, which was not fully involved in the decision making process

Disproportionate amount of workload if a limited number of regional ethics committees will have to review all multi-centre clinical trials

Inability of national or regional ethics committee to monitor all sites physically

Other:

1. (18) Have you ever been part of the team of researchers or investigators for a multi-centre clinical trial that has been reviewed earlier by a common/ designated/ national or regional ethics committee *Mark only one oval.* *

Yes

No

1. (18) (a) If 'YES', in previous question, what was the subsequent step

(If 'NO' kindly skip this question) *

*Mark only one oval.*

The common/ designated/ central ethics committee recommendations sufficed for clearance from ethics at your institute through waiver. Your IEC reviewed matters of local socio-cultural relevance.

The common/ designated/ central ethics committee did not make recommendations on matters related to local socio-cultural contexts, which required a subsequent expedited review from ethics at your institute

The common/ designated/ central ethics committee recommendations required a subsequent full review from ethics at your institute, resulting in two separate full reviews

28. (19) In your opinion, what should be the responsibilities of an IEC for review of a protocol that was earlier reviewed by a common/ designated/ national or regional ethics committee (multiple responses allowed) *

*Tick all that apply.*

Reviewing the clinical trial design and methods of a multi centre clinical trial study protocols and ancillary documents

Overseeing informed consent procedures

Review of adverse events

Causality assessment of serious adverse events

Recommendation of compensation for trial related injury/ death

Ensuring compliance with ethics guidelines and regulations

Monitoring of the respective IEC's participating site of multi centre clinical trials

Engaging with the regulator for submission of SAE related information

Review of local socio-cultural aspects of a clinical trial

Managing conflicts of interest – including MOUs coverage standardization

Addressing Data sharing, ownership of materials and data, intellectual property rights (IPRs), and joint publications.

Other:

*Skip to section 5 (Thank you)*

# Thank you

We thank you for participating in this survey and for your invaluable

contributions, which would be acknowledged anonymously in the final manuscript.. Your feedback will help us understand the current practices and challenges faced by researchers and ethics committee members in evaluating multi-centre clinical trial protocols and monitoring such clinical trials. Please feel free to reach out to the study team if there are any queries.

Please note that this form was meant to be filled in if your primary role is that of a researcher/ investigator in a multi-centre clinical trial. However, if your primary role for the purpose of this survey is that of a ethics committee member, then please use https://forms.gle/y4Kwqo7VzaGDScyk8

## Supplementary Material 2

Google form questionnaire to Ethics Committee Members

**Ethics Review of Multi-Centre Clinical Trials: Ethics Committee Member Perspective**

**Trial Methodology Research by Indian Clinical Trial And Education Network**

**(INTENT)**

Investigators and Contact Information

Dr Aparna Mukherjee (*Principal investigator*), Clinical Studies and Trials Unit, Division of Development Research, Indian Council of Medical Research, New Delhi. (aparna.sinha.deb@icmr.gov.in)

Dr Jerin Jose Cherian (*Co-Investigators*), Clinical Studies and Trials Unit, Division of Development Research, Indian Council of Medical Research, New Delhi

(cherian.jj@icmr.gov.in)

Dr Gunjan Kumar (*Co-Investigators*), Clinical Studies and Trials Unit, Division of Development Research, Indian Council of Medical Research, New Delhi

(gunjank.hq@icmr.gov.in)

**Problem Statement:** Multicentre clinical research are most useful in situations where we need to recruit patients in large numbers, for rare diseases or want to have better generalisable results. However they have various challenges in its planning and conduct. Ethics review of multicentre studies are known to be cumbersome, since the current accepted method is to obtain ethics clearance separately from individual study site. In the interest of improving the Indian clinical research ecosystem, there are various other methods to streamline ethics clearance which are being explored in this survey.

**Invitation to participate in the survey:** You are being invited to participate in this survey to invite your perspectives on ethics review processes. Your expertise in the ethics review of multi-centre clinical trials will prove extremely useful to understand the current challenges and solutions. This is part of a trial methodology research conducted by ICMR's INTENT team in the study titled “Ethics Review of Multi-centre Clinical Trials: An Indian Perspective”. The INTENT program of ICMR is positioned to conduct large multi-centre clinical trials to explore solutions for diseases of national health priority. Streamlining the ethics review process will help INTENT by reducing redundant processes and avoiding delays.

Please note that this form is meant to be filled in if your primary role is that of an Indaian ethics committee member in a multi-centre clinical trial. However, if your primary role for the purpose of this survey is that of a researcher/ investigator, then please use - https://forms.gle/K7d3xxvtsY6iaium6

The survey will take less than fifteen minutes.

**Goal of the survey:** This study aims to understand the perspectives of ethics committee members and researchers regarding ethics review processes in multi centre clinical trials. Your participation in this survey to clearly understand the Indian system for ethics evaluation of multi-centre clinical trials will be invaluable. Before agreeing to participate in this study, it is important that you read and understand the following information.

**Objectives:**

This survey has three objectives:

1. To understand the perspectives of ethics committee members regarding the review of multi-centre trials
2. To understand the challenges faced by researchers in obtaining approval from ethics committees for multi-centre trial **(**[**different survey**](https://forms.gle/K7d3xxvtsY6iaium6)**)**
3. To find out the possible solutions to streamline the ethics review of multi-centre clinical trials

**Study design:** This is a brief, anonymous, national-level, cross-sectional survey administered using Google Forms. We are inviting members of ethics committees, affiliated with diverse types of institutions and organizations from India. We will also reach out to researchers to understand their perspectives on these matters using a separate survey.

**Consent:** We thank you for agreeing to participate in this survey. Your feedback will help us understand the current practices and challenges faced by ethics committee members and researchers in evaluating multi-centre clinical trial protocols and monitoring such clinical trials. Please answer the following questions to the best of your knowledge. The survey will take less than ten minutes. Filling up this survey form will be considered as your consent to use the information provided. There are no identifiers **(no details of participants are collected - including email address)** that are being collected in the survey, and responses will be analysed in a pooled manner.

**Acknowledgement of contribution:** The results of this survey will be used to improve clinical trial ecosystems by streamlining the ethics review process. We will acknowledge the participants of the survey in the manuscript if allowed and the data gathered from this survey will be anonymized before it is published. Please be aware that you will not be receiving any other compensation for participating in this survey.

**Conflict of Interest:** The members of our research team declare that they have no conflict of interest and will receive no financial benefits from any source based on the findings of this survey. Our study does not receive any sponsorship or funding from any source.

**Questions About the Study:** If you have questions about this research study, please contact any of the investigators mentioned earlier.

* Indicates required question

# Participant details

1. (1) Are you an ethics committee member who has reviewed multi centre clinical

trials? *

If you are an ethics committee member as well as a researcher, and wish to share both perspectives, then please fill the other survey form as well intended for the use of researchers - available at the end of the survey.

*Mark only one oval.*

Yes

No *Skip to section 5 (****Thank you****)*

# Participant details

This section will collect general information about the participant and their institution. No identifiers will be collected.

1. (2) Nature of your affiliated institution * *Mark only one oval.*

Public sector teaching hospital

Private sector teaching hospital

Public sector non-teaching hospital

Private sector non-teaching hospital

Industry/ CRO

Independent (Standalone) research institute

Independent (Standalone) ethics committee

Other:

1. (3) Which Indian state is your affiliated institution located in * Dropdown

*Mark only one oval.*

1. (4) Which of the following is the registration status of your institutional ethics committee (IEC ) Mark only one oval *

Dropdown

Both DHR and CDSCO registered

CDSCO registered but not DHR registered

DHR registered but not CDSCO registered

Neither DHR nor CDSCO registered

I don't know

1. (5) How many years of experience do you have in reviewing multi-centre clinical trials as an ethics committee member? * Dropdown

*Mark only one oval.*

1-5 years

5-10 years

10-15 years

>15 years

1. (6) Since 2019, how many multi-centre clinical trials have you reviewed as an ethics committee member? * Dropdown

*Mark only one oval.*

1-5

5-10

10-20

>20

1. (7) In your opinion, how effective is your IEC in fulfilling its role in review and monitoring of a multi centre clinical trial? *

*Mark only one oval.*

- - 1. Very effective
    2. Somewhat effective
    3. Neutral
    4. Not very effective
    5. Not at all effective
    6. I don't know

1. (8) In your opinion, what are the main challenges faced by your IEC for effective review and monitoring of multi centre clinical trials? (multiple responses allowed) *

*Tick all that apply.*

- 1. Limited resources (e.g., funding, personnel)
  2. Insufficient training or expertise
  3. High workload leading to delay
  4. Lack of standardized procedures
  5. Difficulty in harmonizing decisions with other ethics committees
  6. No challenges

Other:

1. (9) What are the common issues discussed during the ethics review of a multicentre clinical trial. (Multiple responses allowed)

*Tick all that apply.*

Reviewing the clinical trial design and methods of a multi centre clinical trial study protocols and ancillary documents

Overseeing informed consent procedures

Review of adverse events

Causality assessment of serious adverse events

Recommendation of compensation for trial related injury/ death

Ensuring compliance with ethics guidelines and regulations

Monitoring of the respective IEC's participating site of multi centre clinical trials

Engaging with the regulator for submission of SAE related information

Review of local socio-cultural aspects of a clinical trial

Managing conflicts of interest – including MOUs coverage standardization

Addressing Data sharing, ownership of materials and data, intellectual property rights (IPRs), and joint publications.

Other:

10. (10) How would you rank the average time taken by your Ethics committee to review multi-centre clinical trial protocols? *

*Mark only one oval.*

a)Very efficient (Consistently meets timelines without significant delays)

b)Generally efficient (Usually meets timelines, occasional minor delays that do

no impact the trial)

c)Adequate (Meets timelines, but sometimes delays occur which have minor

impact on the trial)

d)Inadequate ( Frequent delays impacting timelines of the trial significantly)

e)Not aware

# For Ethics committee members

This section of the survey tries to understand the perspectives of ethics committee members regarding the review of multi-centre trials. We also explore potential solutions to streamline the ethics review of multi-centre clinical trials.

1. (11) Are you aware of the draft guidelines for Common Ethics Review of Multicentre Research by ICMR that is currently available for public consultation inviting comments from relevant stakeholders?

*Mark only one oval.*

Yes

No

1. (11) a. If ‘YES’ to the previous question, Are there any specific areas or issues unaddressed in the ICMR guidelines? (If ‘NO’ to previous question, kindly skip)
2. (12) Do you think that a guideline for ethics review of multicentre trials would aid ethics committees in their decision making? *

*Mark only one oval.*

Yes

No

Not Sure

1. (12) a. Do you feel special training should be provided to ethics committee members to review multicentre trials? *

*Mark only one oval.*

Yes

No

Maybe

1. (13) Although it is NOT mandatory for investigators to provide extracts of Ethics Committee (EC) decisions from other participating centres for multicentre trials, do you believe it should become a regular practice and part of the guideline?

*Mark only one oval.*

Yes

No

1. (14) As one of the many sites in a multi centre clinical trial, what are the challenges you have faced as a member of the IEC in reviewing the master protocol (multiple responses allowed) *

*Tick all that apply.*

Conflicting recommendations / decisions across different IECs

Inability to modify master protocol of the multi centre clinical trial

No formal mechanism to engage with other sites' IECs to share information regarding SAE, monitoring, protocol deviations etc.

Inability to monitor other sites in the clinical trial

Other:

1. (14) (a) Please describe the challenges based on the response(s) chosen in question 14 (In up to 200 words) *
2. (15) As the approving IEC of one site in a multi-centre clinical trial, do you communicate with IECs of other participating sites *Mark only one oval.*

No

Yes

I don't know

1. (15) (a) If 'Yes' in previous question, what topics do you find necessary to communicate (multiple responses allowed) (If 'NO' Kindly skip this question)

*Tick all that apply.*

Adverse events

Serious adverse events

Progress of enrolment

Protocol deviation

Compensation for trial related injury

Other:

1. (16) How often have you come across conflicting ethics committee recommendations/decisions for a multi-centre clinical trial? *

*Mark only one oval.*

Always

Often

Rarely

Never

1. (17) In your opinion, which of the following processes would most effective in improving the ethics review of a multi-centre clinical trial protocol (choose only one most appropriate response) *Mark only one oval.*

Common ethics review (when representative IEC members of all the participating centres in a multi-centre clinical trial convene together to review the protocol)

Designated ethics committee (when one of the participating centres takes the lead role to review the protocol of a multi-centre clinical trial and their recommendations are sufficient for the IECs of remaining trial sites to allow the conduct of the trial after review of ethics aspects with local socio-cultural relevance)

National or regional ethics committee (when a dedicated ethics committee is constituted to review multi-centre clinical trial protocols and their recommendations are sufficient for the IECs of remaining trial sites to allow the conduct of the trial after review of ethics aspects with local socio-cultural relevance)

Full ethics review from each participating site's IEC, as is practiced currently

1. (17) (a) Please justify the response chosen in question 17 (mandatory, in up to

200 words) *

1. (17) (b) From the following challenges, which are the ones that will be faced in case of implementation of **common ethics review** (multiple responses allowed)

[Common ethics review = when representative IEC members of all the participating centres in a multi-centre clinical trial convene together to review the protocol]

*Tick all that apply.*

Logistic issues to plan/ conduct the meeting

Disagreements between members are difficult to resolve

Every trial will end up having a new ethics committee

Difficulty to identify the chairperson and member secretary

Monitoring of the trial will continue to be the responsibility of the site IEC, which was not fully involved in the decision making process

Other:

1. (17) (c) From the following challenges, which are the ones that will be faced in * case of implementation of **designated ethics review** (multiple responses allowed)

[Designated ethics committee = when one of the participating centres takes the lead role to review the protocol of a multi-centre clinical trial and their recommendations are sufficient for the IECs of remaining trial sites to allow the conduct of the trial after review of ethics aspects with local socio-cultural relevance]

*Tick all that apply.*

There is no clear process/ criteria to identify the designated ethics committee

Decision making can seem unilateral and might not be agreeable to the local IECs

Monitoring of the trial will continue to be the responsibility of the site IEC, which was not fully involved in the decision making process

Inability of designated ethics committee to monitor all sites physically

Other:

25. (17) (c1) In your opinion, if a **designated ethics committee** were to review the protocol of a multi-centre clinical trial, what would be the criteria based on which such a designation would be assigned to a particular IEC

[Designated ethics committee = when one of the participating centres takes the lead role to review the protocol of a multi-centre clinical trial and their recommendations are sufficient for the IECs of remaining trial sites to allow the conduct of the trial after review of ethics aspects with local socio-cultural relevance]

IEC of the institute of lead PI can be designated

IEC with maximum experience in review of clinical trials, especially multi-centre clinical trials can be designated

IEC with closest date available amongst the various sites can be designated

IEC scoring highest in an objective evaluation tool, such as the WHO Tool for

Benchmarking Ethics Oversight of Health-Related Research can be designated

IEC with the least rejection rate can be designated

Other:

26. (17) (d) From the following challenges, which are the ones that will be faced in * case of implementation of **national or regional ethics committee** (multiple responses allowed)

[National or regional ethics committee = when a dedicated ethics committee is constituted to review multi-centre clinical trial protocols and their recommendations are sufficient for the IECs of remaining trial sites to allow the conduct of the trial after review of ethics aspects with local socio-cultural relevance]

*Tick all that apply.*

There is no clear process to constitute the national or regional ethics committee

Decision making can seem unilateral and might not be agreeable to the local IECs

Monitoring of the trial will continue to be the responsibility of the site IEC, which was not fully involved in the decision making process

Disproportionate amount of workload and untoward delays if a limited number of regional ethics committees will have to review all multi centre clinical trials Inability of national or regional ethics committee to monitor all sites physically

Other:

1. (18) Have you ever received for review the protocol of a multi-centre clinical trial that has been reviewed earlier by a common/ designated/ national or regional ethics committee *Mark only one oval.*

Yes

No

1. (18) (a) If 'YES', in previous question, what was the subsequent step ?

(If 'NO' kindly skip this question) *Mark only one oval.*

The common/ designated/ central ethics committee recommendations sufficed for clearance from ethics at your institute through waiver. Your IEC reviewed matters of local socio-cultural relevance.

The common/ designated/ central ethics committee did not make recommendations on matters related to local socio-cultural contexts, which required a subsequent expedited review from ethics at your institute

The common/ designated/ central ethics committee recommendations required a subsequent full review from ethics at your institute, resulting in two separate full reviews

29. (19) In your opinion, what should be the responsibilities of an IEC for review of a protocol that was earlier reviewed by a common/ designated/ national or regional ethics committee (multiple responses allowed)

*Tick all that apply.*

Reviewing the clinical trial design and methods of a multi centre clinical trial study protocols and ancillary documents

Overseeing informed consent procedures

Review of adverse events

Causality assessment of serious adverse events

Recommendation of compensation for trial related injury/ death

Ensuring compliance with ethics guidelines and regulations

Monitoring of the respective IEC's participating site of multi centre clinical trials

Engaging with the regulator for submission of SAE related information

Review of local socio-cultural aspects of a clinical trial

Managing conflicts of interest – including MOUs coverage standardization

Addressing Data sharing, ownership of materials and data, intellectual property rights (IPRs), and joint publications.

Other:

*Skip to section 5 (Thank you)*

# Thank you

We thank you for participating in this survey and for your invaluable contributions which would be acknowledged anonymously in the final manuscript. Your feedback will help us understand the current practices and challenges faced by ethics committee members and researchers in evaluating multi-centre clinical trial protocols and monitoring such clinical trials. Please feel free to reach out to the study team if there are any queries.

Please note that this form was meant to be filled in if your primary role is that of an ethics committee member in a multi-centre clinical trial. However, if your primary role for the purpose of this survey is that of a researcher/ investigator, then please use <https://forms.gle/K7d3xxvtsY6iaium6>

## Supplementary Material 3

STROBE Checklist for Cross-Sectional Survey

|  | **Item No** | **Recommendation** | **Page No** |
| --- | --- | --- | --- |
| **Title and abstract** | 1 | (*a*) Indicate the study’s design with a commonly used term in the title or the abstract | 2 |
|  |  | (*b*) Provide in the abstract an informative and balanced summary of what was done and what was found | 2 |
| **Introduction** | | | |
| Background/rationale | 2 | Explain the scientific background and rationale for the investigation being reported | 3 |
| Objectives | 3 | State specific objectives, including any prespecified hypotheses | 4 |
| **Methods** | | | |
| Study design | 4 | Present key elements of study design early in the paper | 4 |
| Setting | 5 | Describe the setting, locations, and relevant dates, including periods of recruitment, exposure, follow-up, and data collection | 4,5 |
| Participants | 6 | (*a*) Give the eligibility criteria, and the sources and methods of selection of participants | 4,5 |
| Variables | 7 | Clearly define all outcomes, exposures, predictors, potential confounders, and effect modifiers. Give diagnostic criteria, if applicable | NA |
| Data sources/ measurement | 8* | For each variable of interest, give sources of data and details of methods of assessment (measurement). Describe comparability of assessment methods if there is more than one group | 7,8 |
| Bias | 9 | Describe any efforts to address potential sources of bias | 16 |
| Study size | 10 | Explain how the study size was arrived at | 4,5 |
| Quantitative variables | 11 | Explain how quantitative variables were handled in the analyses. If applicable, describe which groupings were chosen and why | 7 |
| Statistical methods | 12 | (*a*) Describe all statistical methods, including those used to control for confounding | 7 |
|  |  | (*b*) Describe any methods used to examine subgroups and interactions | 7 |
|  |  | (*c*) Explain how missing data were addressed | 8 |
|  |  | (*d*) If applicable, describe analytical methods taking account of sampling strategy | 7 |
|  |  | (*e*) Describe any sensitivity analyses | NA |
| **Results** | | | |
| Participants | 13* | (a) Report numbers of individuals at each stage of study - eg numbers potentially eligible, examined for eligibility, confirmed eligible, included in the study, completing follow-up, and analysed | 8 |
|  |  | (b) Give reasons for non-participation at each stage | 8 |
|  |  | (c) Consider use of a flow diagram | NA |
| Descriptive data | 14* | (a) Give characteristics of study participants (eg demographic, clinical, social) and information on exposures and potential confounders | 8 |
|  |  | (b) Indicate number of participants with missing data for each variable of interest | 8 |
| Outcome data | 15* | Report numbers of outcome events or summary measures | 8-10 |
| Main results | 16 | (*a*) Give unadjusted estimates and, if applicable, confounder-adjusted estimates and their precision (eg, 95% confidence interval). Make clear which confounders were adjusted for and why they were included | NA |
|  |  | (*b*) Report category boundaries when continuous variables were categorized | NA |
|  |  | (*c*) If relevant, consider translating estimates of relative risk into absolute risk for a meaningful time period | NA |
| Other analyses | 17 | Report other analyses done—eg analyses of subgroups and interactions, and sensitivity analyses | 9 |
| **Discussion** | | | |
| Key results | 18 | Summarise key results with reference to study objectives | 12-14 |
| Limitations | 19 | Discuss limitations of the study, taking into account sources of potential bias or imprecision. Discuss both direction and magnitude of any potential bias | 16 |
| Interpretation | 20 | Give a cautious overall interpretation of results considering objectives, limitations, multiplicity of analyses, results from similar studies, and other relevant evidence | 17-18 |
| Generalisability | 21 | Discuss the generalisability (external validity) of the study results | 17 |
| **Other information** | | | |
| Funding | 22 | Give the source of funding and the role of the funders for the present study and, if applicable, for the original study on which the present article is based | 19 |

## Supplementary Material 4

The major challenge encountered by most researchers while obtaining clearance for multi-centre protocols was varying timelines across different Institutional Ethics Committees (IECs), reported by 83(78%) researchers and conflicting recommendations or decisions across IECS cited by 76 (72%) of researchers, suggesting a lack of standardization in the process which can add to the delay in timelines. Additional concerns included inconsistent IEC capacity and documentation burden.

It was noted that EC members face a number of difficulties while reviewing the master protocol for multi-centre studies. The lack of a formal mechanism to share critical information to participating ECs, was cited by 80 (59%) EC members. 76(56%) expressed that inability to modify the protocol and difficulty in monitoring other sites within the trial 71(52%) was significant challenge, details of which are described in the table below

Challenges in ethics review of multicentre trials: protocol submission through EC review

|  | | Number of responses; n(%)† |
| --- | --- | --- |
|  |  | Researchers Total (N)=106 |
| Challenges faced as a researcher/Investigator in obtaining ethics clearance for protocols of multicentre trials | Varying timelines across different IECs | 83(78%) |
|  | Conflicting recommendations/decisions across different IECs | 76(73%) |
|  | Inconsistent capacity across different IECs | 59(56%) |
|  | Documentation Burden | 47(44%) |
|  | Others *Limited understanding of multi-centre studies* | 1(1%) |
|  | | EC Members  Total (N)=136 |
| Challenges faced as an EC member in reviewing the Master Protocol | No formal mechanism to engage with other sites' IECs to share information regarding SAE, monitoring, protocol deviations etc. | 81(60%) |
|  | Inability to modify master protocol of the multicentre trial | 76(56%) |
|  | Inability to monitor other sites in the clinical trial | 71(52%) |
|  | Conflicting recommendations/decisions across different IECs | 54(40%) |
|  | Others *(Variable stringency in review processes among participating centres)* | 1(1%) |

† Multiple responses allowed from respondents

## Supplementary Material 5

Distribution of Ethics Committee Members’ Preferences for Ethics Review Processes by Institutional Affiliation (Public vs. Private)

|  | EC members  (N=136) | | | Researcher  (N=106) | | |
| --- | --- | --- | --- | --- | --- | --- |
| Affiliation | Private (n=95) | Public (n=41) |  | Private (n=47) | Public (n=59) |  |
| Individual Ethics Review (status quo) | 27(28) | 15(37) | P=0.69 | 15(32) | 7(12) | P=0.07 |
| Designated Ethics Review | 21(22) | 7(17) |  | 13(28) | 19(32) |  |
| Joint Ethics Review | 30(32) | 14(34) |  | 12(25) | 17(29) |  |
| National Ethics Review | 17(18) | 5(12) |  | 7(15) | 16(27) |  |

Values are expressed as number(%).

No statistically significant difference was observed in the preference for the ethics review process between the ethics committee members and researchers belonging to the private and public section

## Supplementary Material 6

Preferences for Ethics Review Processes Among Ethics Committee Members and Researchers Stratified by Years of Experience

|  | EC member (N=136) | | | Researcher (N=106) | | |
| --- | --- | --- | --- | --- | --- | --- |
| Years of experience  (coordinating/ conducting/ reviewing) | 1-10 years  (n=105) | >10 years  (n=31) |  | 1-10 years  (n=74) | >10 years  (n=32) |  |
| Individual Ethics Review (status quo) | 31(30) | 11(35) | P=0.37 | 12(11) | 10(9) | P=0.37 |
| Designated Ethics Review | 22(21) | 6(19) |  | 24(23) | 8(8) |  |
| Joint Ethics Review | 32(31) | 12(39) |  | 21(20) | 8(8) |  |
| National Ethics Review | 20(19) | 2(6) |  | 17(16) | 6(6) |  |

Values are expressed as number (%). No statistically significant difference was observed in the preference for the ethics review process between ethics committee members and researchers with 1–10 years and those with >10 years of experience.

## Supplementary Material 7

Preferences for Ethics Review Processes Among Ethics Committee Members and Researchers Stratified by Number of MCT Experience

|  | EC member (N=136) | | | Researcher (N=106) | | |
| --- | --- | --- | --- | --- | --- | --- |
| No of MCT Experience  (coordinated/ conducted/ reviewed) | 1-10 trials  (n=104) | >10 trials  (n=32) |  | 1-10 trials  (n=98) | >10 trials  (n=8) |  |
| Individual Ethics Review (status quo) | 27(20) | 15(11) | P=0.11 | 19(18) | 3(3) | P=0.44 |
| Designated Ethics Review | 21(15) | 7(5) |  | 31(30) | 1(1) |  |
| Joint Ethics Review | 37(27) | 7(5) |  | 26(25) | 3(3) |  |
| National Ethics Review | 19(14) | 3(2) |  | 22(21) | 1(1) |  |

Values are expressed as number (%). No statistically significant difference was observed in the preference for the ethics review process between ethics committee members and researchers with experience in 1–10 MCTs compared to those with >10 MCTs.

## Supplementary Material 8

Criteria for Selecting a Designated Ethics Review Committee:


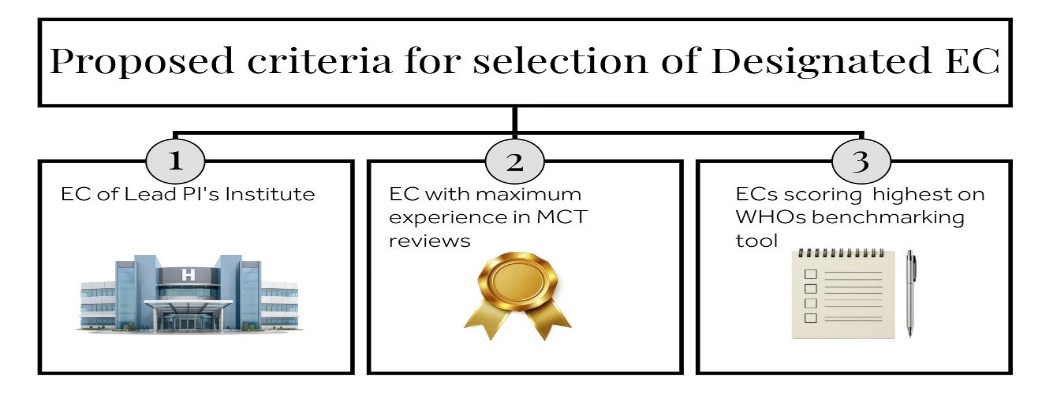


Respondents were asked how a designated ethics committee (EC) should be identified for multi-centre trials. The majority of both researchers and EC members agreed upon three key criteria that could guide this selection:

1. EC of the institute of lead PI can be designated; 2. EC with maximum experience in review of clinical trials, especially MCTs can be designated; 3. EC scoring highest in an objective evaluation tool, such as the WHO Tool for Benchmarking Ethics Oversight of Health-Related Research can be designated.

## Supplementary Material 9

Responses regarding study protocol that were previously reviewed by a joint/designated/national ethics committee were analysed to understand what subsequent step was followed. A small proportion of respondents i.e. 26(25%) researchers and 25(18%) EC faced such situations, whereas the majority did not come across such protocols for review nor were part of such studies. When asked about the subsequent steps followed at the participating IECs during instances where a MCT protocol previously reviewed either by a joint/designated/national ethics review committee, the majority of researchers 14(54%) stated that despite the initial recommendations, the protocol required a subsequent re-review, resulting in *two separate reviews*. In contrast, only 3(12%) of EC members stated that a subsequent full review at each local IEC was mandated in such instances. Majority EC members 12(48%) stated that joint/designated/national EC recommendations sufficed for approval from local IECs, which reviewed only matters of local socio cultural relevance with 10(40%) indicating that expedited review from their local IECs were only matters related to local socio-cultural context. The table below highlights the responsibilities of an IEC during a re review of a Multi-centre protocol previously reviewed either by a joint/designated/ national ethics review committee.

IEC Responsibilities During Re-review of Multi-centre Protocols

|  | Number of responses; n(%)† | |
| --- | --- | --- |
|  | Researchers Total (N)=106 | EC Members  Total (N)=136 |
| Ensuring compliance with ethics guidelines and regulations | 71(67%) | 93(68%) |
| Reviewing the clinical trial design and methods of a multi-centre clinical trial study protocols and ancillary documents | 50(47%) | 91(67%) |
| Overseeing informed consent procedures | 65(61%) | 91(67%) |
| Review of adverse events | 66(62%) | 9O(66%) |
| Recommendation of compensation for trial related injury/ death | 61(58%) | 79(58%) |
| Causality assessment of serious adverse events | 57(54%) | 78(57%) |
| Monitoring of the respective IEC's participating site of multi-centre clinical trials | 63(59%) | 73(54%) |
| Engaging with the regulator for submission of SAE related information | 49(46%) | 66(49%) |
| Review of local socio-cultural aspects of a clinical trial | 64(60%) | 84(62%) |
| Managing conflicts of interest – including MOUs coverage standardization | 56(53%) | 72(53%) |
| Addressing Data sharing, ownership of materials and data, intellectual property rights (IPRs), and joint publications. | 49(46%) | 67(49%) |

^†^ Multiple responses allowed from respondents

## Supplementary Material 10

A tabular summary of the advantages of various ethics review processes based on the responses from EC members and researchers are provided below

Advantages of various ethics review processes

|  |  | **Ethics Review Processes** | | | | | | | |
| --- | --- | --- | --- | --- | --- | --- | --- | --- | --- |
| **Categories** | **Sub-Categories (Advantages**^¥^**)** | **Joint Ethics Review** | | **Designated Ethics Review** | | **Regional/National Ethics Review** | | **Individual Site Ethics Review** | |
|  |  | **EC Members (n=40)** | **Researchers (n=29)** | **EC Members (n=20)** | **Researchers (n=27)** | **EC Members (n=13)** | **Researchers (n=13)** | **EC Members (n=31)** | **Researchers (n=10)** |
| Local context | Addresses site specific requirements/ Equal representation/ Local contextual insights/ Address Diversity | 4 | 2 | 3 | 1 | 1 | 0 | 18 | 9 |
| Consistency | Standardisation/ streamlining/ uniform processes | 5 | 6 | 7 | 8 | 7 | 6 | 2 | 0 |
|  | Collaborative decision making | 20 | 9 | 1 | 6 | 2 | 0 | 0 | 0 |
| Efficiency | Efficiency and reduces delay | 9 | 12 | 8 | 11 | 3 | 4 | 3 | 1 |
| Expertise | Experienced reviewers/ expert inclusion | 2 | 0 | 1 | 1 | 0 | 3 | 0 | 0 |

^¥^ Categories were derived through coding of qualitative responses explaining reasons for preference for each ethics review model. n represents the number of respondents who provided a justification for that model; hence the number of responses varies across columns
